# Three-dimensional-printed customized prosthesis for pubic defect: prosthesis design and surgical techniques

**DOI:** 10.1186/s13018-020-01766-8

**Published:** 2020-07-13

**Authors:** Yuqi Zhang, Li Min, Minxun Lu, Jie Wang, Yitian Wang, Yi Luo, Yong Zhou, Hong Duan, Chongqi Tu

**Affiliations:** 1 Department of Orthopedics, West China Hospital, Sichuan University, No. 37 Guoxuexiang, Chengdu, 610041 Sichuan People’s Republic of China; 2 Bone and Joint 3D-Printing & Biomechanical Laboratory, Department of Orthopedics, West China Hospital, Sichuan University, No. 37 Guoxuexiang, Chengdu, 610041 Sichuan People’s Republic of China

**Keywords:** 3D-printed, Prosthesis, Type III hemipelvectomy, Design solution, Surgical technique

## Abstract

**Background:**

This study is to describe the detailed design and surgical techniques of three-dimensional (3D)-printed customized prosthesis for pubic bone defect.

**Patients and methods:**

Five patients under type III resections were included in this study. Based on radiography data, 3D pelvic model was established and virtual surgery was simulated. Detailed anatomy data were measured including the size and arc of normal pubis, the size of residual bone in acetabular side. Different fixation ways were considered according to shape of defect. After features modification and porous structure design, prostheses were fabricated. The osteotomy guides and plastic models were used during surgery.

**Result:**

Of 5 cases, the prostheses consist of the type with stem (3, 60%) and the type without stem (2, 40%). Mean follow-up period was 13.6 months (range, 8-24 months). For partial pubis removed cases, the mean length and width of narrowest part of normal superior pubis were 13.19 mm (range, 12.51-14.12 mm) and 7.80 mm (range, 7.18-8.26 mm) respectively. Mean arc of normal pubis was 2.71 rad (range, 2.66-2.73 rad). For the entire pubis resection cases, the mean diameter of narrowest parts and length of normal superior pubis were 11.52 mm (range, 11.13-11.91 mm) and 64.78 mm (range, 63.46-66.09 mm), while the diameter of narrowest part and length of normal inferior pubis were 7.37 mm (range, 7.20-7.54 mm) and 86.43 mm (range, 84.28-88.57 mm). Mean length and arc of intramedullary stem was 20 mm (range, 18-21 mm) and 2.7 rad. Mean screw holes number was 6.3 (range, 6-7) while ultimate screws number in surgeries was 4.3 (range, 4-5). Porous structure with 600-μm-pore size and 70% porosity was applied in parts of contact with residual bone.

**Conclusion:**

3D-printed customized prostheses could be a feasible option to reconstruct bone defect after type III resection. The design of 3D-printed customized prostheses is a multi-step process which is based on strict anatomic measurement.

## Background

Pelvectomy and defect reconstruction after resection are common ways to treat pelvic tumors. Both resection and construction are challenging for surgeons due to the complexity and irregularity of pelvis. According to Enneking’s classification of pelvectomy [1], the type III resection refers to the partial removal of the pubis or the pubis from the pubic symphysis to the lateral obturator. But type III resection is uncommon, accounting for only about 11% [2–4] in pelvic resection. Although partial defects involving region III were reconstructed, most pure type III resections were usually not reconstructed because the weight-bearing axis, which goes through the proximal femur, acetabulum, sciatic buttress, and spine, is preserved [4–6].

However, more and more studies confirmed that type III resection without reconstruction can cause a series of complications. Hernia was frequently reported as a late complication in patients without reconstruction after type III resections [7, 8]. And reconstruction provides an anchor for mesh and suture attachments, which adds to the integrity of pelvic floor soft tissue reconstructions. That is the reason why some surgeons who do not reconstruct the bone defect still try pelvic floor repair [9]. Besides, according to Tile [10], the anterior pelvic ring structure accounts for 40% of the stability of the entire pelvic ring, and the posterior ring structure accounts for 60%. Furthermore, study shows that patients developed stress fractures without reconstruction after type III internal hemipelvectomy because the residual pelvic bones become unstable and distorted during walking and running [6]. So, the integrity of the anterior pelvic ring should not be ignored, although the continuity of the weight-bearing axis was preserved. Thus, reconstruction after type III resection is accepted by more and more people.

At present, allograft, mesh, and artificial ligament are the most common reconstruction methods after type III resection [11, 12]. Although allograft could provide a bony reconstruction, high operative infection rate was frequently reported by some researches [2]. Researches showed that the infection rate of allograft after type III internal hemipelvectomy is as high as 20% [11, 13]. Soft tissue reconstruction like mesh and the artificial ligament is easy and convenient, but the mechanical stability of the pelvis is ignored which results in changes of pelvic structure and mechanics causing complications like acetabular shift and sacroiliitis. In addition, the prosthesis is also a choice to reconstruct pelvis and has good initial stability, early weight bearing and relatively rapid restoration of function, it has not been applied in reconstruction after type III resection.

With progress in three-dimensional (3D)-printing technology, 3D-printed customized prosthesis become a more accepted choice for irregular bone defects, such as pelvic defect. Nowadays, 3D-printed customized prostheses in the pelvis are mainly used in hemipelvectomy including type I and type II resection with or without partial pubis [14, 15]. For pure type III defect reconstruction, there is no relative research reported so far.

In this study, we designed 3D-printed customized prostheses for type III defect of the pelvis, and performed implantation successfully. Favorable clinical outcomes were observed. And the detailed design of prosthesis and surgical techniques was described.

## Method

### Patients

**Fig. 1:**
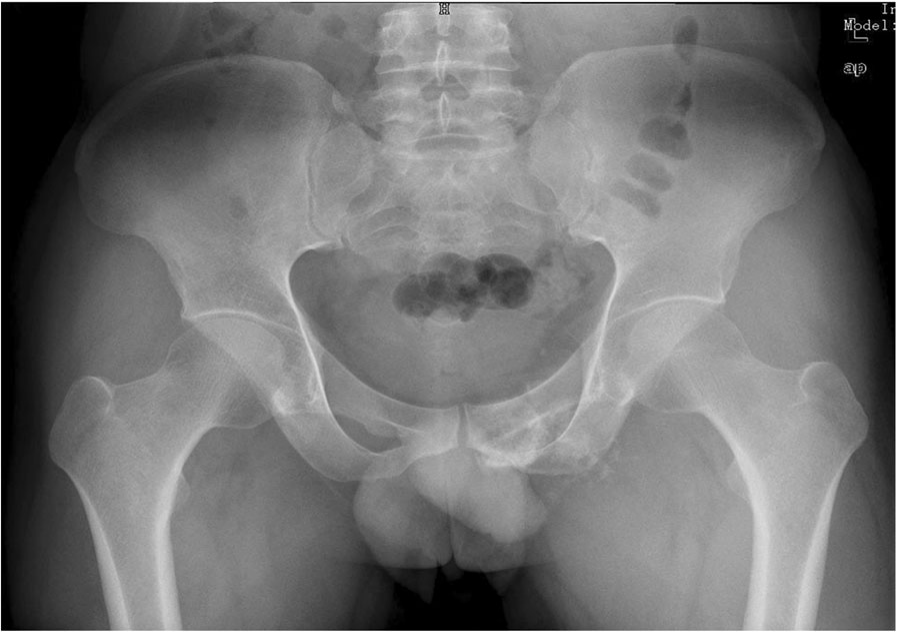
Pelvic X-ray in a patient with an chondrosarcoma (after biopsy) involving the superior and inferior pubis

Between June 2017 and February 2019, 5 patients received type III en bloc resection and three dimensional printed prosthetic reconstruction in our institution. There were 3 males and 2 females with a mean age of 36.6 years (range, 26-46 years) at the time of surgery. 3 patients underwent one ramus of pubis resection while 2 patients underwent both pubic rami resection. The characteristics of the patients are summarized in Table 1. Every patient underwent pelvic X-ray, magnetic resonance imaging (MRI), 3D computerized tomography (3D CT), single-photon emission computed tomography (SPECT) or positron emission tomography/computerized tomography (PET/CT) and preoperative biopsy (Fig. 1).

**Table 1:** The characteristics of the patients

Patientno.	Age (years)	Gender	Tumor location	Pathological type	Prosthesis type	Follow-up (months)
1	37	Male	Entire pubis	Chondrosarcoma	No stem	8
2	26	Female	Entire pubis	Chondrosarcoma	No stem	10
3	44	Male	Pubic superioris	Chondrosarcoma	With stem	17
4	30	Male	Pubic superioris	Chondrosarcoma	With stem	19
5	46	Female	Pubic superioris	Chondrosarcoma	With stem	24
Mean	36.6	-	-		-	15.6

### Anatomy data measurement

**Fig. 2:**
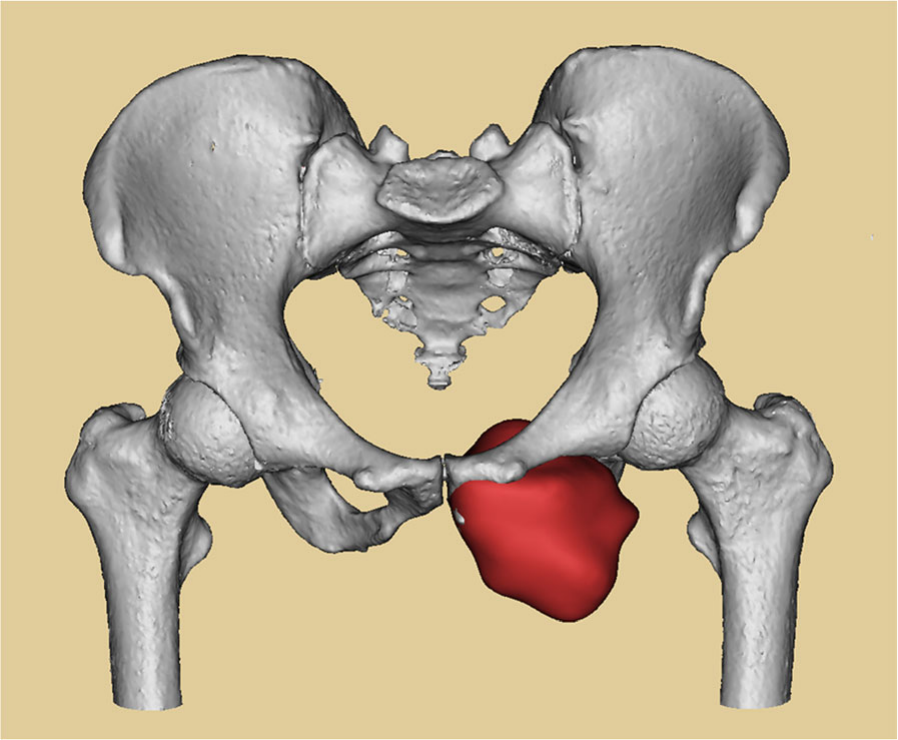
Pelvis (white) and tumor model (red) modeled with 3D CT and MRI

3D CT files of all patients were imported to the Mimics V20.0 software (Materialise Corp., Leuven, Belgium) to build three-dimensional models of tumor and pelvis and measure pubic data. The size of normal superior pubic narrowest part and arc of normal superior pubis were measured in three cases whose superior pubes were resected. While the size of normal superior and inferior pubis, the thickness and height of internal superior acetabular bone was measured in two cases whose entire pubis were resected. The margin of tumor was determined by the combination of X-ray, MRI, SPECT, and 3D CT on the basis of 3D model. Then the curative margin was obtained to determine the tumor resection part and residual bone part (Fig. 2).

### Prosthesis design and fabrication

**Fig. 3:**
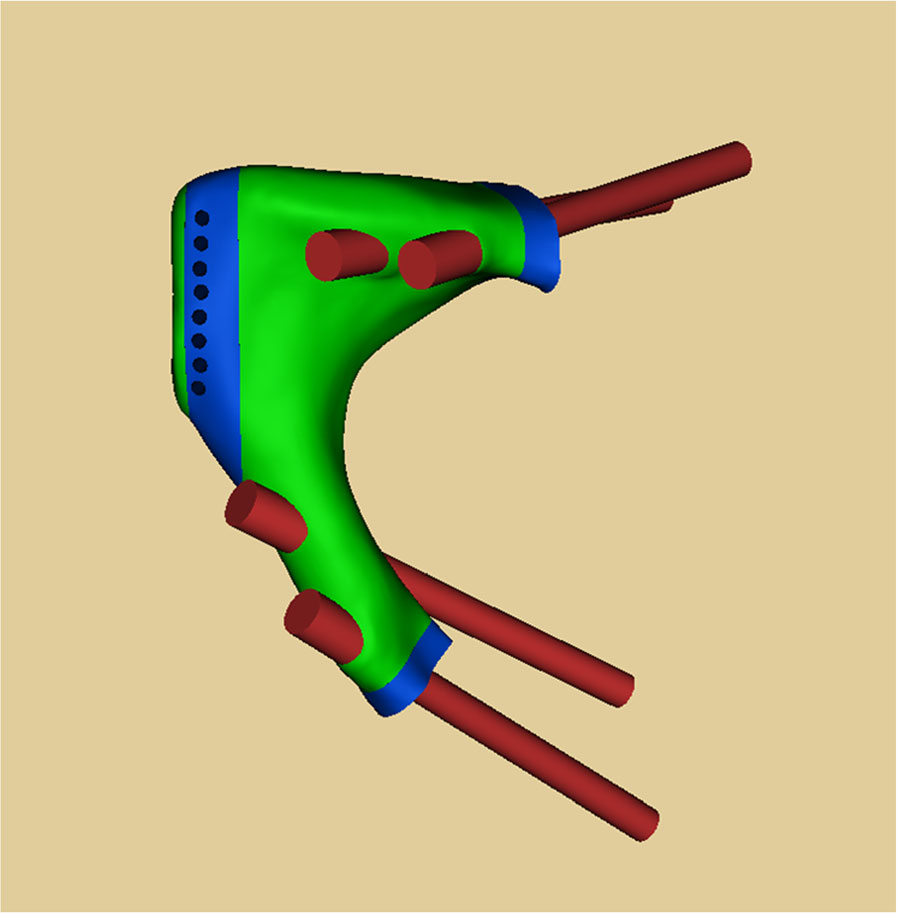
The prosthesis was composed of solid structure and porous structure, and matched bone defect. Two screws fixed the prosthesis to anterior column of acetabulum, and two screws fixed the prosthesis to sciatic bone

**Fig. 4:**
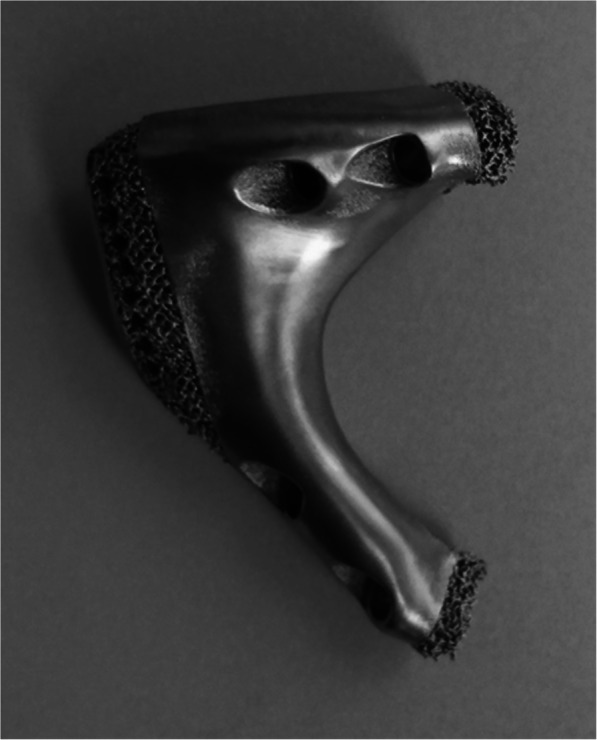
Prosthesis fabricated with 70% porosity and 600-μm-pore size by electron beam melting technique

According to the shape of the tumor resection part and residual bone part, the preliminary shape of the prosthesis was determined by mirroring normal corresponding part in the Geomagic Studio software (Geomagic Inc., Morrisville, United States). And the osteotomy guides were designed. After that, specific features were added to the prosthesis. Next, removing unnecessary features and smoothing the surface of prosthesis. In the end, a porous structure was separated and generated in the Magics V20 software (Materialise Corp., Leuven, Belgium). The osteotomy guides and prostheses were saved as stereolithography (STL) files, and the size of prostheses was measured in Mimics. Prosthesis was fabricated by electron beam melting technology (ARCAM Q10plus, Mölndal, Sweden) (Figs. 3 and 4).

### Surgical techniques

**Fig. 5:**
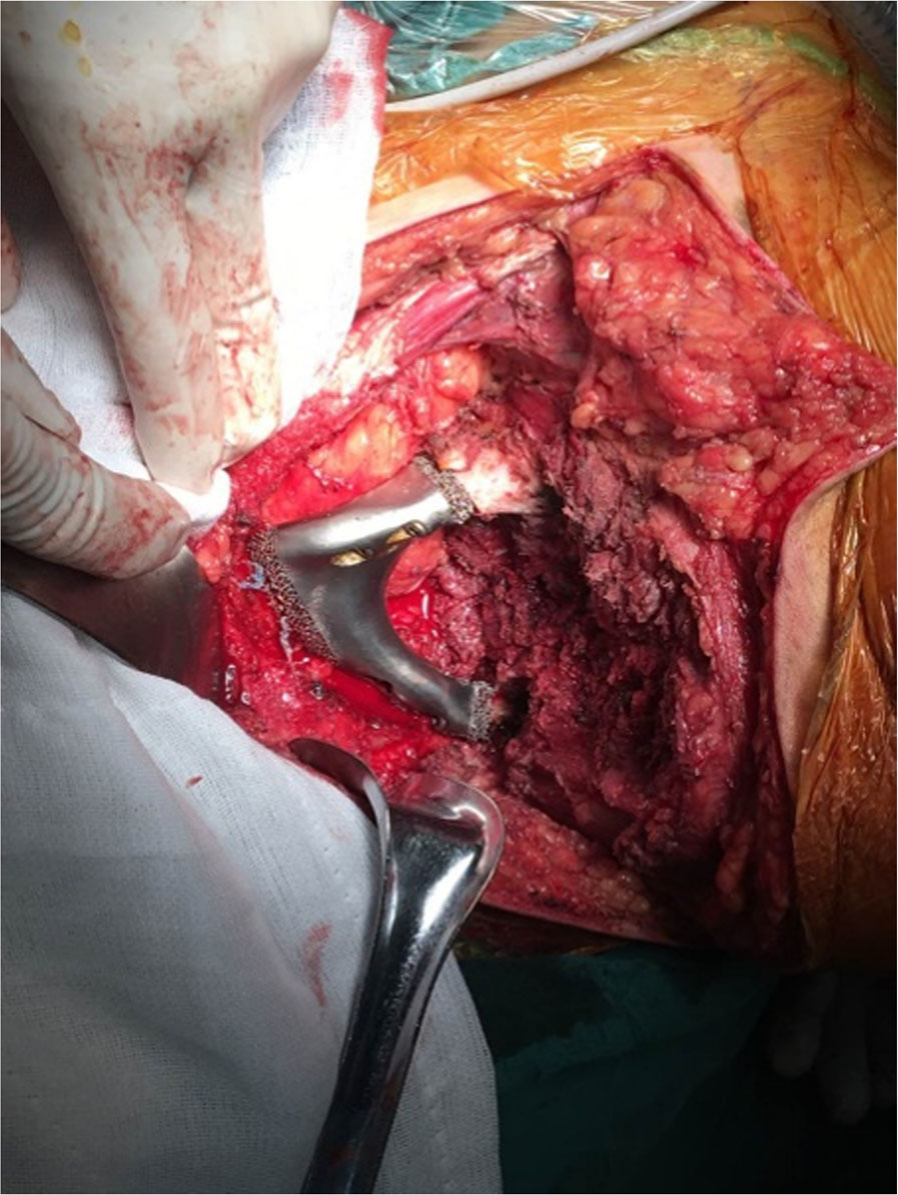
The prosthesis was implanted and fixed with screws

All surgeries were performed by the same senior surgeon (Chongqi Tu). The patients were placed in the oblique supine lithotomy position. Ilioinguinal incision was mostly used. Osteotomy was performed with the help of osteotomy guides and specific bone structures. Besides the tumor resection, subfascial dissection with the removal of all of the muscles in the compartment was done. At the level of the bone osteotomy, the muscles were severed. Then the plastic trial model with the same proportion of the prosthesis was used first to confirm a perfect match between the defect and the prosthesis. The next step is the prosthesis implantation with fixation by screws, plates, sutures, or intramedullary stem. Some muscles were reconstructed either before stitching (Fig. 5).

### Postoperative management

For patients who received prosthesis with stem replacement, bed rest and movement limiting of the affected limb for 3-4 weeks were undertaken. Meanwhile, the flexion, extension, and internal rotation of the hip joint were executed with adductor relaxation and contraction. Passive adduction and external rotation were allowed within 30° before week four, and gradually changed to positive movement at week five to prevent the failure of adductor reconstruction. Walking with crutches began at week five and weight bearing of the affected limb increased gradually. Two months postoperatively, walking on flat ground without crutches was allowed.

Three-four days bed rest was recommended for those patients who received prostheses without stem replacement. The movement of the hip joint and adductor relaxation and contraction was undergone with the passive abduction and external rotation during the first week. And during the second week, walking with crutches with gradually increasing weight bearing was undergone. The positive abduction and external rotation began at the third or fourth week. Patients could walk on flat ground without crutches a month later after surgery.

All patients underwent postoperative X-ray to assess prosthetic position, and were evaluated regularly (monthly in first 3 months and then trimonthly) with physical examination, X-ray, tomosynthesis-shimadzu metal artifact reduction technology (T-SMART) of pelvis. CT of the chest was used to determine metastasis. Osseointegration was evaluated by T-SMART. Functional outcome was assessed with Musculoskeletal Tumor Society (MSTS) score (Rating scale is based on 7 items including pain, range of motion, strength, joint stability, joint deformity, emotional acceptance, and overall function. Each item is scored from 0-5 with a maximum possible score of 35.). Complications were recorded.

### Results

Prostheses were designed based on the anatomy data we measured. For partial pubis removed cases, the mean length and width of the narrowest part of normal superior pubis were 13.19 mm (range, 12.51-14.12 mm) and 7.80 mm (range, 7.18-8.26 mm) respectively. Mean arc of normal pubis was 2.71 rad (range, 2.66-2.73 rad). For the entire pubis resection cases, the mean diameter of narrowest parts and length of normal superior pubis were 11.52 mm (range, 11.13-11.91 mm) and 64.78 mm (range, 63.46-66.09 mm), while the diameter of narrowest part and length of normal inferior pubis were 7.37 mm (range, 7.20-7.54 mm) and 86.43 mm (range, 84.28-88.57 mm). Then, the mean thickness and height of the residual bone in internal superior acetabulum were 12.82 mm (range, 12.71-12.93 mm) and 26.81 mm (range, 26.57-27.05 mm). Detailed measurement data were summarized in Table 2.

**Table 2:** The anatomy data of patients

Patient no.	Superior acetabular bone/mm		Superior pubis/mm		Inferior pubis/mm		Narrowest part of superior pubis			Arc of pubis/rad
	Thickness	Height	Diameter of narrowest part	Length	Diameter of narrowest part	Length	Area/mm^2^	Length/mm	Width/mm	
1	12.71	27.05	11.13	63.46	7.20	84.28	-*	-	-	-
2	12.93	26.57	11.91	66.09	7.54	88.57	-	-	-	-
3	-	-	-	-	-	-	98.75	12.51	7.95	2.73
4	-	-	-	-	-	-	115.62	14.12	8.26	2.66
5	-	-	-	-	-	-	91.59	12.93	7.18	2.73
mean	12.82	26.81	11.52	64.78	7.37	86.43	101.97	13.19	7.80	2.71

*“-” means unmeasured

**Fig. 6:**
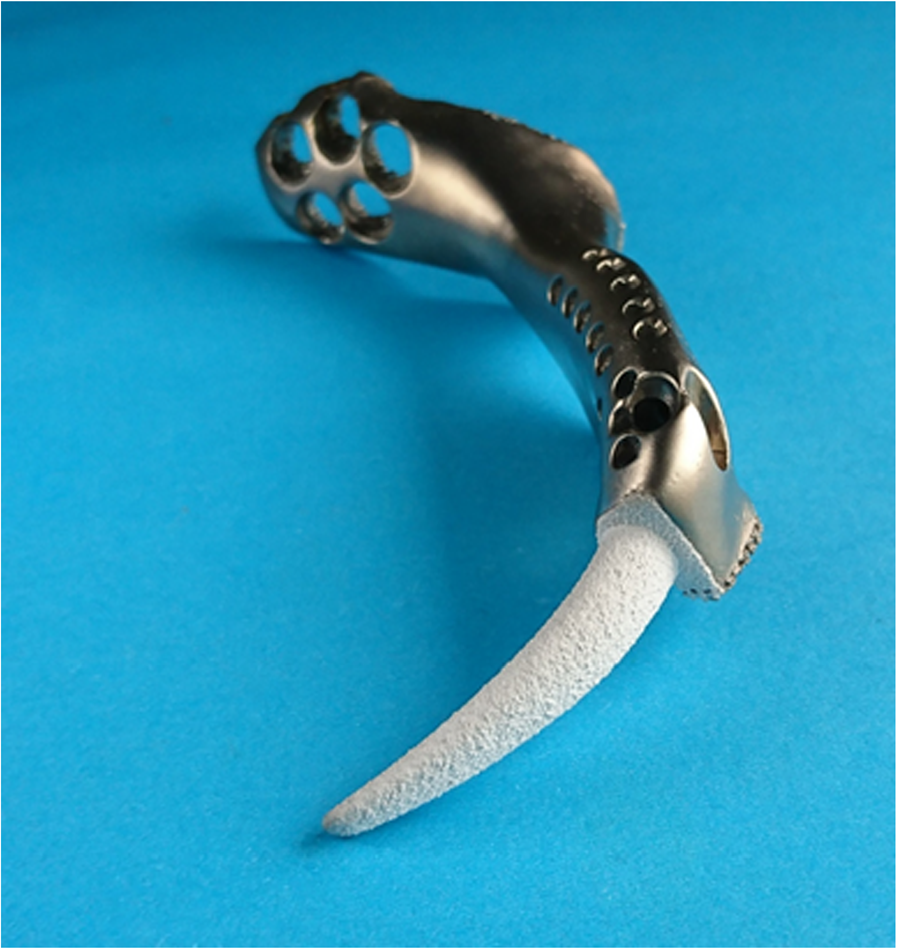
Prosthesis with stem

**Fig. 7:**
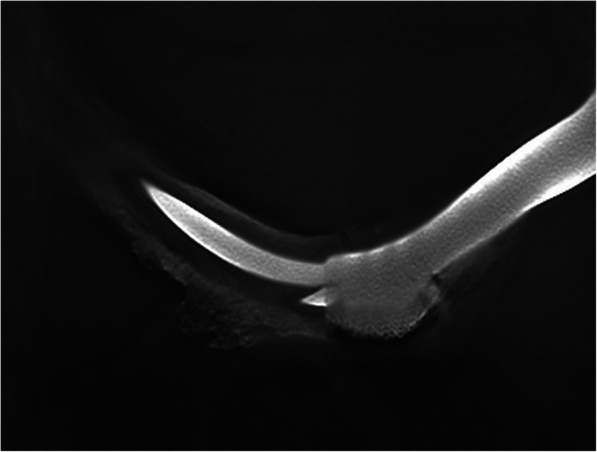
Six months after operation, T-SMART showed fretting wear appeared around prosthetic stem

Prostheses were composed of solid and porous structures. Porous structure with 600-μm-pore size and 70% porosity was applied in parts of contact with residual bone. According to the resection extent, we designed the prosthesis with stem (Fig. 6): The superior ramus of pubis and partial pubic symphysis were reconstructed. Assisted plate, pubic stem, and screws inserting to residual pubic medullary cavity were applied. The out face of plate and lower face of ramus were porous structures and screw holes were added on plate. Six months follow-up results showed that fretting wear appeared around the prosthetic stem (Fig. 7). Based on the first kind of prosthesis, the prosthesis without stem (Fig. 4) was designed: Entire pubis and the pubic symphysis were reconstructed. The prosthesis was designed like the normal pubis which contained symphysis, pubis-ischium and pubis-ilium interfaces. Symphysis interface was smooth and fixed by rivet string through a string of stitch holes parallel to the interface while the part around stitch holes was designed to be porous structures. Pubis-ischium and pubis-ilium interfaces were porous structures and screw holes were added.

Mean length and arc of the intramedullary stem was 20 mm (range, 18-21 mm) and 2.7 rad. Mean screw holes number was 6.3 (range, 6-7) while ultimate screws number in surgeries was 4.3 (range, 4-5). Mean thickness of plate was 8.0 mm (range, 7.5-8.5 mm). Screw holes diameter was 53 mm and screws diameter was 50 mm. Mean intraoperative time was 294 min (180 to 430 min) and blood loss was 1680 ml (300 to 3700 ml).

Preoperative averaged MSTS score was 31 (range, 29-33).

**Fig. 8:**
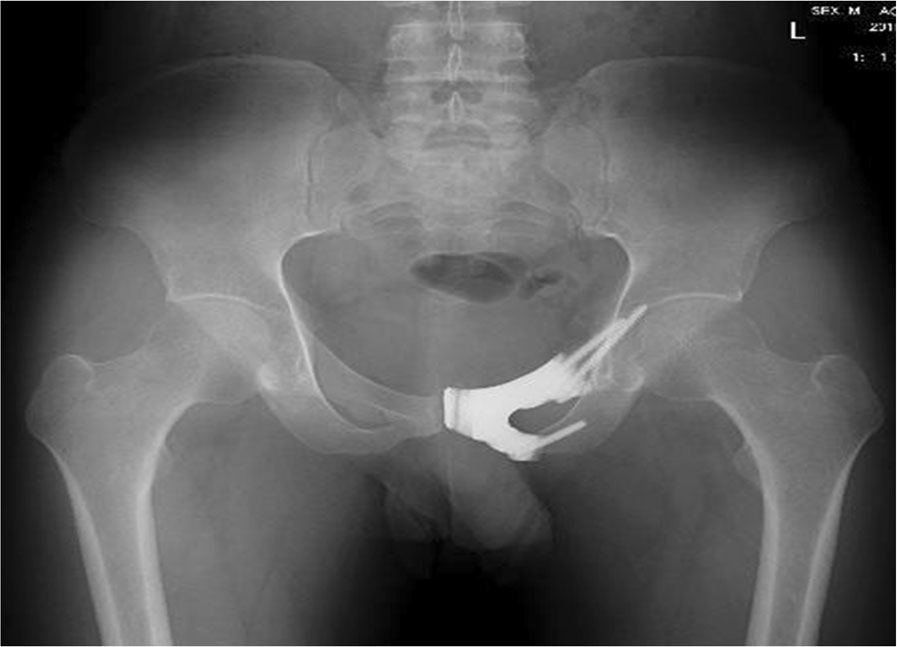
Two months after the operation, X-ray showed well alignment of prosthesis

**Fig. 9:**
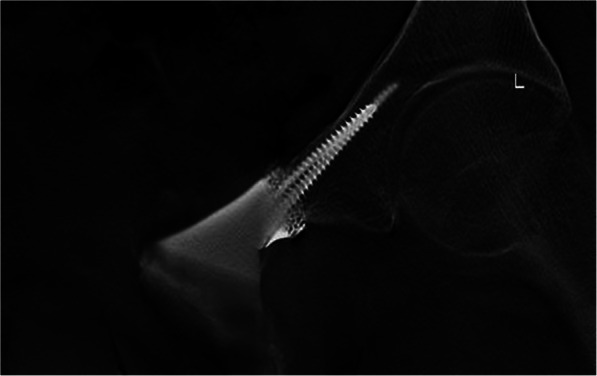
Three months after the operation, T-SMART showed preliminary osseointegration

Mean follow-up period was 13.6 months (range, 8-24 months). Averaged MSTS score was 30 (range, 29-31). No significant difference compared with preoperative MSTS score. No dislocation, intraoperative bleeding, and postoperative infection and distant metastasis were observed during follow-up. T-SMART showed absence of interfacial gap between the prosthesis and bone 6 months postoperatively (Figs. 8 and 9).

## Discussion

Up to now, allograft, mesh repair, and artificial ligament repair are the main choices for reconstruction after type III resection [11, 12, 16]. Recent reconstruction ways after type III resection were listed in Table 3. Allograft can provide a pelvic bony reconstruction and restore the stability of the pelvis. Compared with non-reconstruction, better function outcomes and pain relief were observed in studies [11]. However, infection, nonunion, and fracture are the main complications. Karim, S.M.’s research reported that complications of reconstruction for type III defect with allograft included infection in two, symptomatic hernia in one, hip instability in one, dislocated total hip arthroplasty in one, and graft failure in one among 14 patients. Also, the most commonly used bone for graft is the fibula [16], whose shape cannot match the defect perfectly. On the other hand, soft tissue reconstruction is also acceptable. Artificial ligament (ligament advanced reinforcement system, LARS), marlex mesh, and soft tissue flap were commonly used relatively [3, 9, 17]. Although these ways could reconstruct the inferior abdominal wall defect and prevent the hernia effectively, the anatomic position of pelvis cannot be restored and the anterior pelvic ring cannot be maintained which leads to hip joint displace and sacroiliac joint stress concentration.

**Table 3:** Recent reconstruction ways after type III resection

First author	Year	Type of reconstruction	Number of patients	Follow-up time	MSTS
Laurel, A.	1989	No reconstruction	12	0.75-15 years	N/A*
Timothy Jorden	2002	Marlex mesh	9	0.75-12 years	N/A
Reddy, S.S.	2012	Marlex mesh	8	9.5 years(average)	N/A
Courtney E. Sherman	2011	No reconstruction	8	N/A	N/A
Nikolaos Arkoulis	2012	No reconstruction	1	N/A	N/A
Jungo Imanishi	2015	No reconstruction, fascia lata	2	N/A	100
Rosyane, R. D. F.	2015	Fibular graft	2	N/A	N/A
		No reconstruction	3	N/A	N/A
Albert, H.	2015	Mesh, soft tissue flap	14	N/A	N/A
Karim, S.M.	2015	Allograft	5	0.58-6 years	N/A
Zhang J	2018	LARS ligament	25	1.33-4 years	88
RĂZVAN ENE	2018	No reconstruction	1	N/A	N/A

*N/A means not available

Recently, 3D-printed customized prostheses are increasingly being used in the pelvic defect reconstruction and have been reported with good early results [18]. But 3D-printed customized prostheses have not been applied in defect reconstruction after type III resection. In this study, detailed anatomy data was measured and two kinds of 3D-printed customized prostheses were designed based on the extent of tumor invasion.

First of all, the feature of the preliminarily designed prosthesis needs modification. Suture holes along prosthesis were added to reattach muscles and fascia, such as adductor longus and brevis, internal oblique, and external oblique muscles which are important for preventing hernia. Then, the main body was divided into solid and porous structures in order to reduce prosthesis weight, guarantee prosthesis strength, and enhance bone ingrowth. Previous studies showed that the porous structures (300 to 800-μm-pore size and 70% porosity) at the interface can enhance bone ingrowth [19–23]. So, porous structures with porosity of 70% and pore size of 600 μm were applied in this study, and osseointegration was well observed in patients.

Prostheses fixation, including fixation in pubic symphysis side and fixation in acetabular side, is very important for the stability and osseointegration. (1) Fixation in acetabular side: The prostheses were fixed by screws and plates. The number, diameters, directions, and depth of screws were designed according to the detailed anatomy data of the residual bone. In the first kind of prosthesis reconstruction, we designed screw holes and a plate for fixation. Plate was fixed on the medial side of the pelvis by screws which went through superior side of acetabulum and another screw fixed prosthesis to the inferior pubis. In addition, there were screws inserting to the residual pubic medullary cavity to prevent the prosthesis from separating after restoration. In the second kind of prosthesis reconstruction, screw holes were designed in a special direction to pass the screws from the anterior and medial side of the acetabulum. (2) Prostheses fixation in the pubic symphysis side is significant for the whole pelvis. Considering ways to reconstruct pubis in other hemipelvic prostheses, we designed the prosthesis initially with intramedullary stem to maintain pelvic stability. However, there were signs of fretting wear around the prosthetic stem in follow-up. And we found fretting wear appeared in partial patients who underwent hemipelvic prostheses replacement with rigid fixation like plates or screws. In addition, the pelvis is a closed ring connected by two joints with low mobility: pubic symphysis and sacroiliac joint. So, we considered that sacroiliac joint with low mobility can affect the rigid fixation of the pubic symphysis. In other words, if the sacroiliac joint is intact or still retains some mobility, the pubis symphysis reconstruction also should be movable. Conversely, if sacroiliac joint lost mobility, the pubis symphysis reconstruction should be rigid. Otherwise, these two joints were contradictory which leads to fretting wear of prosthesis or sacroiliitis. Thus, we improved the previous design and tried to reconstruct pubis symphysis to get some mobility instead of rigid fixation. Modified prosthesis was tied to the contralateral pubis with rivet string and the interface was smooth to simulate normal symphysis. And follow-up shows no dislocation and displacement.

What is more, the design of 3D-printed customized prostheses should be flexible. Different fixation ways could be combined to deal with complex defects. Assisted plate can be designed when prosthesis cannot be fixed by screws or the intramedullary stem, while screws and intramedullary stem also could be combined if necessary. And the principle for screw holes design is that the direction of screw holes should be convenient for the surgeon to insert screws and should avoid other important structures like acetabulum.

On the other hand, intraoperative precise osteotomy, proper endoprosthesis implantation, and correct screws insertion are critical in the operation of 3D-printed prosthetic reconstruction. Osteotomy guides were applied for providing the desired osteotomy plane with less exposure and instrument requiring during operation. Specific anatomy features should be the location point of the guides, such as the angle of the superior and inferior pubis. Additionally, due to complex anatomy of the pelvis and severe displacement of residual pelvic bone after resection, proper placement of prosthesis is technically demanding: (1) plastic trial model should be used to assess whether the prosthesis matches the defect, and plastic trial model should be with a smaller stem or without stem for avoiding affecting the stability of prosthesis and provide convenience for implantation; (2) bone surface in contact with prosthesis should remove partially cortex to expose the cancellous bone to promote bone ingrowth; (3) conformation of well placement should be done before inserting screws by checking pelvic continuity near osteotomy plane; (4) the order of prostheses fixation is critical for implantation. For prosthesis with stem, the intramedullary stem should be inserted into the medullary cavity first and then fixing the prosthesis to the acetabular side bone. While for prostheses without stem, fix the prosthesis to the acetabular side bone first and then reconstruct the pubis symphysis.

Our study has some limitations. First, this is a retrospective case series of a small number of patients with a short follow-up, long-term follow-up is required. Second, it is also possible that more complications or problems might arise as long-term follow-up. Third, because of small numbers, different extents of resection, and disease processes, it is difficult to make a control group to compare with. In addition, there is no biomechanical analysis in our study, so finite element analysis should be down in the next step. Therefore, further study should be continued and a multi-institutional study is needed.

## Conclusion

Reconstruction after type III pelvectomy is necessary. 3D-printed customized prostheses could be a feasible option to reconstruct bone defect after type III resection. The design of 3D-printed customized prostheses is a multi-step process that involves measurement, design, manufacture, and surgery. Despite favorable outcomes, we observed some imperfections in preoperative design and surgical application, more works are required in further study.

## Data Availability

The data and materials are available from the medical records department of the West China Hospital. The datasets used and analyzed during the current study are available from the corresponding author on reasonable request.

## Abbreviations

3D: Three dimensional
CT: Computerized tomography
MRI: Magnetic resonance imaging
SPECT: Single-photon emission computed tomography
PET/CT: Positron emission tomography/computerized tomography
T-SMART: Tomosynthesis-shimadzu metal artifact reduction technology
MSTS: Musculoskeletal Tumor Society
